# Primality, Fractality, and Image Analysis

**DOI:** 10.3390/e21030304

**Published:** 2019-03-21

**Authors:** Emanuel Guariglia

**Affiliations:** 1 Department of Mathematics and Applications “R. Caccioppoli”, University of Naples Federico II, 80126 Naples, Italy; emanuel.guariglia@gmail.com; 2 School of Economics, Management and Statistics, University of Bologna, 40126 Bologna, Italy

**Keywords:** binary image, Cantor set, Hénon map, Minkowski island, prime-indexed primes, Ramanujan primes, Primary: 11N05, 62H35, Secondary: 28A80, 37D45

## Abstract

This paper deals with the hidden structure of prime numbers. Previous numerical studies have already indicated a fractal-like behavior of prime-indexed primes. The construction of binary images enables us to generalize this result. In fact, two-integer sequences can easily be converted into a two-color image. In particular, the resulting method shows that both the coprimality condition and Ramanujan primes resemble the Minkowski island and Cantor set, respectively. Furthermore, the comparison between prime-indexed primes and Ramanujan primes is introduced and discussed. Thus the Cantor set covers a relevant role in the fractal-like description of prime numbers. The results confirm the feasibility of the method based on binary images. The link between fractal sets and chaotic dynamical systems may allow the characterization of the Hénon map only in terms of prime numbers.

## 1. Introduction

The distribution of prime numbers has played an important role in mathematics from the beginning. Nevertheless, the structure of prime numbers has represented a big challenge for generations of mathematicians. In particular, algebraic and analytic features related to the distribution of prime numbers can entail several theoretical problems (the Riemann hypothesis, Goldbach’s conjecture, etc.). However, it is not our purpose to investigate the unsolved problems involving prime numbers [1,2].

Prime numbers belong to number theory, that is, pure mathematics. Nevertheless, they can be linked with almost all modern scientific applications, such as those in quantum cryptography [3], biology [4,5], medicine [6], and dynamical systems [7]. Quite recently, considerable attention has been paid to the link between the distribution of prime numbers, fractal geometry, and chaos theory [8,9,10,11]. In particular, the fractal nature of prime-indexed primes (PIPs) has been discussed in [12], where the author has numerically shown the quasi-self similar fractality of finite-differenced PIP sequences. The main result arising from this paper is that order of prime numbers and scale of fractal sets play the same role. In [13] the existence of a link between some prime sequences and the Cantor set is shown. The author investigated Cantor primes, that is, prime numbers *p* such that 1/p belongs to the Cantor set, providing characterization results. The importance and complexity of the prime distribution led to the development of several techniques for the resolution of unsolved problems. In modern number theory many subsets of the prime numbers (twin primes, Ramanujan primes, Chen primes, etc.) play a relevant role. Thus the fractal nature of prime numbers can be partially determined, showing the fractality of these subsets. The main difficulty in carrying out this technique is that the set of prime numbers cannot currently be partitioned into these subsets. However, the gap between prime numbers seems to be the key point for understanding the structure of prime distribution. In fact, in [14] the author has investigated the possibility of expressing gaps between consecutive primes in terms of the prime counting function. In addition, the same author has shown that the distribution of prime numbers has a powerlike behavior, that is, a long-range dependence. This suggests some kind of self-similarity in prime distribution.

The existence of patchiness in prime distribution can be studied by gaps between consecutive primes. The results of Wolf [11,14] indicate that these patches can be expressed in terms of the prime counting function, which allows a self-similar approximation [15]. Investigation into the nature of these gaps may reveal the hidden structure of prime numbers. For instance, the randomness in prime distribution is linked to the Sierpinski gasket in [10]. All these partial results indicate that the behavior of prime numbers resembles the recursive law of a fractal set. Thus this work is intended to motivate further investigation into the chaoticness of prime numbers.

The main purpose of this paper is to shine new light on the hidden structure of the prime distribution through an investigation in fractal geometry. In particular, the key research question is whether or not the coprimality condition, PIPs, and Ramanujan primes have fractal-like behavior. The approach adopted for this study is based on image analysis. In fact, the construction of a Boolean matrix enables us to define the correspondent correlation matrix, which provides the binary image sought. The results resemble well-known fractal patterns. More precisely, the coprimality condition and Ramanujan primes exhibit behavior based on the Minkowski island and Cantor dust, respectively. Furthermore, PIPs and Ramanujan primes are compared and the results discussed. Cattani and Ciancio [16] showed that the distribution of PIPs is similar to the Cantor dust. The Hénon map, which is related to a Cantor-like set, provides an application in chaotic dynamical systems. Thus the Cantor set seems to play a noteworthy and relevant role in the distribution of prime numbers.

The remainder of the paper is organized as follows. Section 2 presents some preliminaries on PIPs, Ramanujan primes, and Rényi dimension. Section 3 outlines the main results of the binary image linked to both the coprimality condition and Ramanujan primes. Furthermore, the comparison between *k*-order PIPs and Ramanujan primes is presented and discussed. In Section 4, the Hénon map provides an application in chaotic dynamical systems. Finally, Section 5 summaries the results and concludes with a suggestion for further investigation.

## 2. Preliminaries

Throughout this paper and unless otherwise specified, *k* and *n* will indicate elements of $N=\left\{1,2,3,\ldots \right\}$. Likewise, α and *x* will always denote real numbers. The greatest common divisor of two integers *a* and *b* is denoted by $\left(a,b\right)$. In addition, *a* and *b* are called coprime (or relatively prime) whenever $\left(a,b\right)=1$. The cardinality of a set *A* will be denoted by #A. For the convenience of the reader and for brevity the preliminaries are reported below without proofs [8,17,18], thus making our exposition self-contained.

### 2.1. Prime Numbers, PIPs, and Ramanujan Primes

Let $D_{n}$ be the set given by

$$D_{n}:=\left\{k\in N:k|n\right\},\;n\geq 1\;.$$

It follows immediately that the elements of $D_{n}$ are simply all the positive divisors of *n*. Any natural number p>1 is called prime if $\#\;D_{p}=2$. For simplicity of notation, let $p_{i}$ (i≥1) stand for the *i*th prime number. Thus the set of prime numbers is defined as follows:

$$P:=\{p_{i}:i\in N\}\;.$$

Therefore P={2,3,5,7,11,…}. Euclid showed that #P=∞ although the distribution of prime numbers within N is still an open problem. The main result is a (statistical) property called the prime number theorem. This involves the prime counting function, π:R→N, defined by

$$\pi \left(x\right)=\#\;P_{x}\;,\;P_{x}:=\left\{p_{i}\in P:p_{i}\leq x\right\}\;,$$

that is, the number of primes less than or equal to a given real number *x*. Note that $P_{x}\;\subseteq \;P$. Gauss and Legendre conjectured that π(x) asymptotically tends to x/logx:

(1) $$\pi \left(x\right)∼\frac{x}{\log x}\;\;\;\mathrm{as}\;\;x⟶+\infty \;.$$

Independently, Hadamard and de la Vallée Poussin definitely showed approximation (1), hence the prime number theorem states that

$$\lim_{x\to \infty }\frac{\pi (x)}{x/\log x}=1\;.$$

Both proofs are based on nonvanishing of the Riemann ζ function on the line σ=1, that is ζ(1+it)≠0 for any real *t*. The introduction of the logarithmic integral Li leads to a better approximation given by

Li(x)∼π(x)asx⟶+∞,

which is the current version of the prime number theorem (see [19] for more details).

As already mentioned in Section 1, the focus of this paper is the joint investigation on two subsets of P (PIPs and Ramanujan primes). According to [8,20] the sequence of PIPs, that is the sequence of primes with a prime index, is the subset of P given by

$$P_{1}:=\left\{p_{p_{i}}\in P:p_{i}\in P\right\}\;.$$

Note that the subset $P_{1}$ can be iteratively generalized as follows:

$$P_{2}:=\left\{p_{p_{p_{i}}}\in P:p_{p_{i}}\in P\right\}\;,$$

and so on. This entails that for each integer k≥1, the set $P_{k}$ of the *k*-order PIPs is immediately built. According to the definition of PIPs it follows that $P_{0}=P$. Therefore $P_{k}$ for k=0,1,2 is given by

$$\begin{array}{c} P=\{2,3,5,7,11,13,17,19,23,29,\ldots \}\;,\\ P_{1}=\left\{3,5,11,17,31,41,59,67,83,109,\ldots \right\}\;,\\ P_{2}=\left\{5,11,31,59,127,179,277,331,431,599,\ldots \right\}\;. \end{array}$$

The definition of PIPs entails $P_{k+1}\;\subset \;P_{k}$ for any nonnegative integer *k*. This is consistent with the following approximation

(2) $$\pi^{2}\left(x\right)∼\frac{x}{\log^{2}x}\;\;\;\mathrm{as}\;\;x⟶+\infty \;,\;\pi^{2}\left(x\right):=\pi \left(\pi \left(x\right)\right)\;,$$

shown by Broughan and Barnett [8]. Note that $\pi^{2}\left(x\right)$ is the number of PIPs not greater than a given real *x*. For any integer n≥1, the *n*th Ramanujan prime is defined as the smallest positive integer $R_{n}$ such that

(3) $$x\geq R_{n}⟹\pi \left(x\right)-\pi \left(\frac{x}{2}\right)\geq n\;.$$

Condition (3) ensures the existence of at least *n* prime numbers in the interval $\left(\frac{x}{2},x\right]$ whenever $x\geq R_{n}$. The minimality entails both that $R_{n}$ is always a prime number and that the interval $\left(\frac{R_{n}}{2},R_{n}\right]$ contains exactly *n* primes [17]. The properties of the gamma function allowed Ramanujan to compute the first five values of $R_{n}$, showing that

$$\pi \left(x\right)-\pi \left(\frac{x}{2}\right)\geq 1,2,3,4,5,\ldots ,\;\mathrm{whenever}\;x\geq 2,11,17,29,41,\ldots ,\;\mathrm{respectively}\;.$$

This implies the existence of $R_{n}$. Hence the sequence of Ramanujan primes is the subset of P given by

$$P_{R}=\left\{2,11,17,29,41,47,59,67,71,97,\ldots \right\}\;.$$

The infinitude of PIPs follows directly from approximation (2). A similar approximation shows the infinitude of Ramanujan primes [18].

### 2.2. Fractality and Rényi Dimension

In the last decades, fractal geometry, which plays an important role in modern mathematics, has attracted much attention from research teams. In recent years, there has been a growing interest in the link between fractal geometry and number theory. Fractal sets can often be defined in number theoretic terms. There are different parameters which can used to characterize any fractal set. In particular, fractal dimension and lacunarity [9,21] allow us to investigate the fractal nature of prime sequences.

Recent research on fractal geometry has often been focused on dynamical systems. In fact, previous studies indicate the close link between fractal sets, strange attractors, and entropy [22]. The literature on these topics shows a variety of approaches. In particular, the joint approach has attracted significant attention from many researchers, becoming very popular. The chaoticity can be described by many parameters. Each of them is an index related to the complexity of the system. In particular, the fractal dimension is a noninteger number which describes the irregular shape of fractal sets. There are several definitions of fractal dimension. Investigation along these lines exceeds the scope of this paper. It suffices for our purposes to assume that the fractal dimension coincides with the box-counting dimension. This involves no loss of generality. Let *A* be a non-empty bounded subset of $R^{n}$ and $N_{\delta (A)}$ the smallest number of δ-boxes needed to cover *A*. The box-counting dimension of *A* is given [21,23] by

(4) $${\mathrm{Dim}}_{\;B}\left(A\right):=\lim_{\delta \to 0}\frac{\log N_{\delta }\left(A\right)}{\log(1/\delta )}\;,$$

whenever the limit (4) exists. Definition (4) entails that the box-counting dimension of *A* is the minimum number of δ-boxes covering the set *A* on a uniform grid. The fractal dimension remains the most important parameter in fractal modeling. The box-counting dimension is strongly linked with the concept of entropy. In fact, let *X* be a discrete alphabet random variable and let N=N(δ) be the total number of δ-boxes with $p_{i}>0$. The Rényi dimension, a parameter which describes the multifractality [23,24], is given by

(5) $$D_{\alpha }\left(X\right):=\frac{1}{\alpha -1}\lim_{\delta \to 0}\frac{\log_{b}\overset{N}{\underset{i=1}{\sum }}p_{i}^{\alpha }}{\log_{b}\delta }\;,\;\alpha \geq 0\;,$$

reduces to the box-counting dimension for α=0. It follows immediately that

$$D_{\alpha }=-\lim_{\delta \to 0}\frac{H_{\alpha }}{\log_{b}\delta }\;,$$

where $H_{\alpha }$ is the Rényi entropy. For a fuller treatment see [24] and the references given there.

Fractal sets are mainly characterized by self-similarity and space-filling properties. The concept of lacunarity is related to this second property. In particular, the lacunarity takes into account the texture of a fractal set and describes the distribution of the gaps (lacunae) within it. High values of lacunarity indicate a large size distribution of gaps, that is, a high degree of gappiness. Therefore, the lacunarity draws attention to the homogeneity of a set [9]. High levels of homogeneity correspond to low values of lacunarity, hence the lacunarity provides a valuable idea of the heterogeneity associated with fractal sets. This parameter can be used to distinguish fractal sets with similar fractal dimensions. In other words, fractal dimension and lacunarity play the same role in fractal geometry that mean and variance play in statistics. Nevertheless, lacunarity has been gaining importance in recent years as independent tool to deal with spatial patterns and is often computed by a gliding box algorithm [25].

## 3. Binary Image and Primality

First and foremost, our investigation into the hidden nature of $P_{1}$ and $P_{R}$ may be carried out by considering them as data sets. The major advantage of this approach is that each data set can be described in terms of entropy. In particular, the concept of information entropy introduced by Shannon [24] allows us to compute the entropy of data sets. The information entropy of a discrete alphabet random variable *X* defined on the probability space $\left(\Omega ,B,P\right)$ is given by

$$H\left(X\right):=-\sum_{i=1}^{N}p_{i}\log_{b}p_{i}\;,\;p_{i}=P\left(X=x_{i}\right)\;.$$

In the previous definition of information entropy, the alphabet size is *N* and the most common logarithmic bases are b=2 and b=e. This approach turned out to be inappropriate, as well as a multifractal analysis based on the Rényi dimension (5). In particular, the value of the information entropy does not give us any further information into the hidden structure of prime numbers. Recently, Cattani and Ciancio proposed a new approach to the investigation into the fractality of PIPs [16]. This paper outlines an empiric study on the similarity between the binary image of the PIP (or prime) distribution and Cantor dust. The proposed method is dot-plot based and provides the binary image of these distributions. We now apply this technique to the coprimality condition.

Let $S_{1}=\left\{i_{1},i_{2},\ldots ,i_{k}\right\}$, $S_{2}=\left\{j_{1},j_{2},\ldots ,j_{k}\right\}$ be two *k*-length ordered sequences of natural numbers. The indicator function for coprimality is defined by the binary map

(6) $$f\;:\;S_{1}\times S_{2}\to U\;,$$

such that U is a k×k matrix, called the correlation matrix for coprimately and defined as $U\left(k\times k\right)=\left\{f\left(i_{m},i_{n}\right)\right\}:=\left\{f_{mn}\right\}$ by

$$f_{mn}:=\left\{\begin{array}{cc} 1\;,\; & \left(i_{m},i_{n}\right)=1\;,\\ 0\;,\; & otherwise\;. \end{array}\right.$$

Note that $\left(i_{m},i_{n}\right)\in S_{1}\times S_{2}$ and 1≤m,n≤k. The correlation matrix U for the coprimality condition is shown in Table 1. Clearly, whenever $S_{1}=S_{2}$, the matrix U is simply the autocorrelation of a *k*-length sequence. The indicator function allows us to identify hidden symmetries between two given sequences. In particular, any element of U can be mapped into black and white pixels as follows:

(7) $$\left\{\begin{array}{c} 1⟶black\;pixel\;,\\ 0⟶white\;pixel\;. \end{array}\right.$$

**Figure 1:**
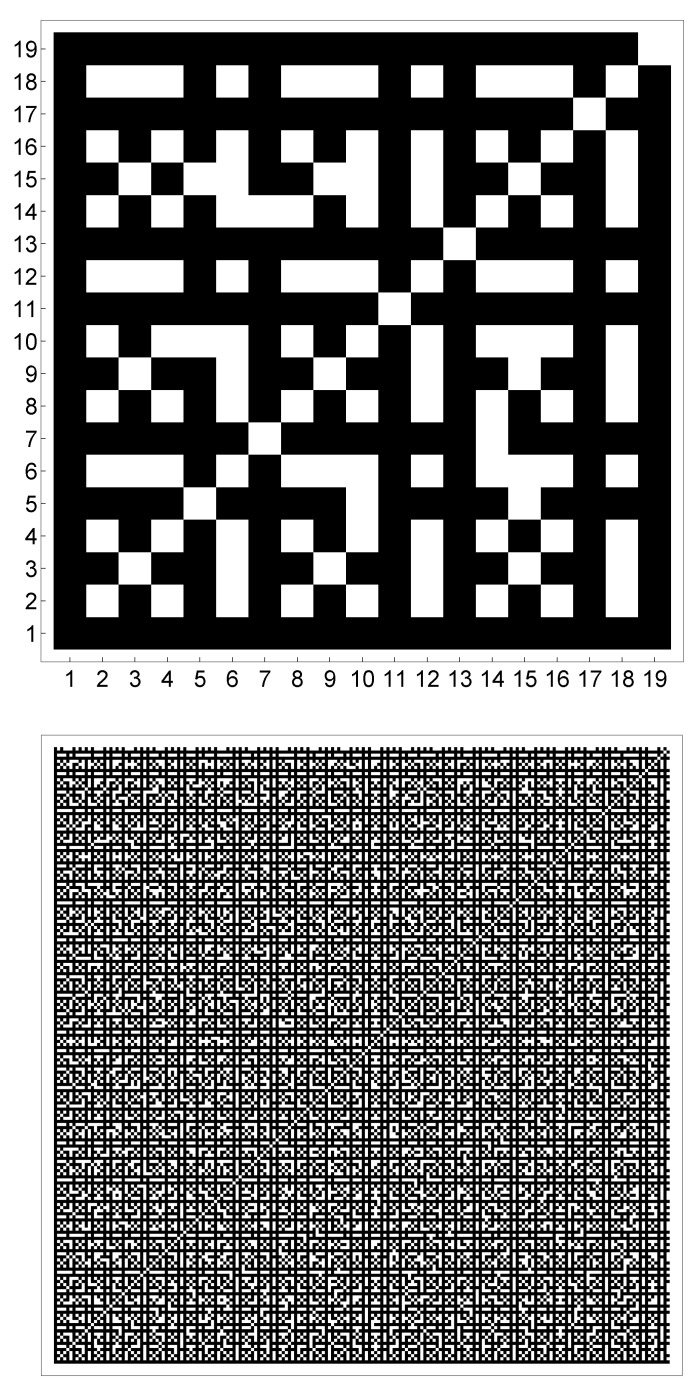
Binary image for the correlation matrix U(k×k) of Table 1 with 1≤k≤19 (**top**) and 1≤k≤200 (**bottom**).

The result of the mapping (7) is a two-dimensional image $I_{k\times k}$, often called a binary image. In image analysis, any image is a set of pixels adjacent to each other. The binary image can be drawn on a white or black support. The white support holds the mapping (7), where foreground pixels are printed in black and background pixels in white (and conversely on a dark support). The composition of the maps *f* and (7) given by

$$S\left(k\right)\times S\left(k\right)\overset{f}{⟶}U\left(k\times k\right)\overset{\left(7\right)}{⟶}I_{k\times k}\;,$$

allows the investigation of the coprimality by fractal geometry. In fact, comparison of Table 1 and Figure 1 (top) shows clearly the presence of the Minkowski island, a variant of the Minkowski curve built on a square. For more details on the Minkowski curve we refer the reader to [26] and the references given there. The lack of some symmetries in Figure 1 (top) might be interpreted as the introduction of randomness in the Minkowski island. Thus the coprimality condition among natural numbers shows a behavior similar to a well-known fractal set. A full discussion requires analytic techniques and lies beyond the scope of this paper.

The statement discussed above is confirmed for higher values. In fact, Figure 1 (bottom) shows the coprimality condition for 1≤k≤200. The behavior is still based on the Minkowski island and is in accordance with theory. In fact, the binary image is symmetric with respect to the main diagonal. This comes from the symmetry of the coprimality condition, that is (m,n)=(n,m) for any m,n∈N.

### 3.1. PIPs and Ramanujan Primes

In view of all that has been outlined in [16], the PIP distribution seems similar to the Cantor dust. The technique based on the binary map (6) can also be applied to the distribution of Ramanujan primes. The correlation matrix $U_{R}$ can easily be computed by definition (3). This give rise to a Boolean table like Table 1. Clearly, the matrix $U_{R}$ is nonzero whenever both the components are Ramanujan primes. According to the notation introduced in definition (6) it is

$$f_{mn}=\left\{\begin{array}{cc} 1\;,\; & \left(i_{m},i_{n}\right)\in P_{R}^{2}\;,\\ 0\;,\; & otherwise\;. \end{array}\right.$$

**Figure 2:**
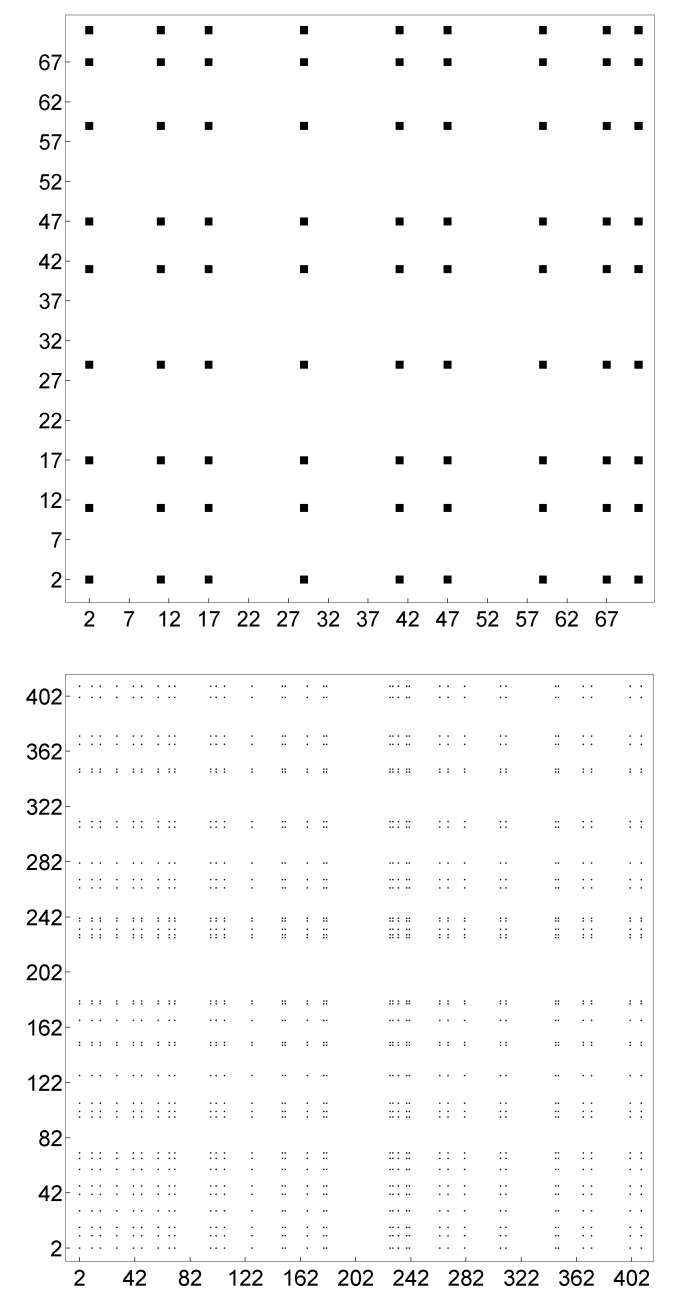
Binary image for the distribution of Ramanujan primes $R_{i}$ among the natural numbers with $2\leq R_{i}\leq 71$ (**top**) and $2\leq R_{i}\leq 409$ (**bottom**).

Note that $S_{1}=S_{2}=P_{R}$ and 1≤m,n≤k. As with the correlation matrix for coprimality, there is no loss of generality in assuming $S_{1}=S_{2}$. Thus the mapping (7) provides the binary image of the Ramanujan primes among the natural numbers, as shown in Figure 2. Like the PIP distribution [16], the distribution of Ramanujan primes looks like the Cantor dust except for some white zones. This lack of symmetry might simply be seen as the presence of randomness in the Cantor dust. The same problem also occurs in the PIP distribution [16]. Let us now define a Cantor prime as any prime number *p* such that 1/p belongs to the (middle-third) Cantor set. Salas has given a characterization of the Cantor set in terms of prime numbers. In particular, he has shown that any prime number p>3 if a Cantor prime if and only if it satisfies an equation which involves both *p* and the order of 3 modulo *p* (see [13] for more details). Accordingly, fractal geometry seems to play a relevant and hidden role in the distribution of prime numbers. However, more study of the issue is required. Further research into the fractal structure of $P_{1}$ and $P_{R}$ should be focused on the main geometric representations of P (sieve of Eratosthenes, Ulam prime spiral, etc.).

### 3.2. Asymptoticity and *k*-Order PIPs

We conclude this section by showing and discussing the recent asymptotic results on PIPs and Ramanujan primes. As mentioned in Section 2, the density of PIPs is $O\left(x/\log^{2}x\right)$. This property is an equivalent of the prime number theorem for the PIP distribution. Broughan and Barnett [8] announced that the same holds for the *k*-order PIPs. Similarly, the results given by Bayless et al. in [20] suggest that

(8) $$\pi^{k}\left(x\right)∼\frac{x}{\log^{k}x}\;\;\;\mathrm{as}\;\;x⟶+\infty \;,$$

where $\pi^{k}\left(x\right)$ denotes the number of *k*-order PIPs less than or equal to a given real number *x*. In addition to this, the counting function $\pi^{k}$ allows explicit bounds. In particular, for any k≥2 there exists a computable $x_{0}\left(k\right)$ such that

$$\frac{x}{\log^{k}x}{\left(1+\frac{1}{\log x}\right)}^{k}{\left(1+\frac{\log\log x}{\log x}\right)}^{k-1}<\pi^{k}\left(x\right)<\frac{x}{\log^{k}x}{\left(1+\frac{1.5}{\log x}\right)}^{k}{\left(1+\frac{1.5\log\log x}{\log x}\right)}^{k-1}\;,\;\;\forall x\geq x_{0}\left(k\right)\;.$$

**Figure 3:**
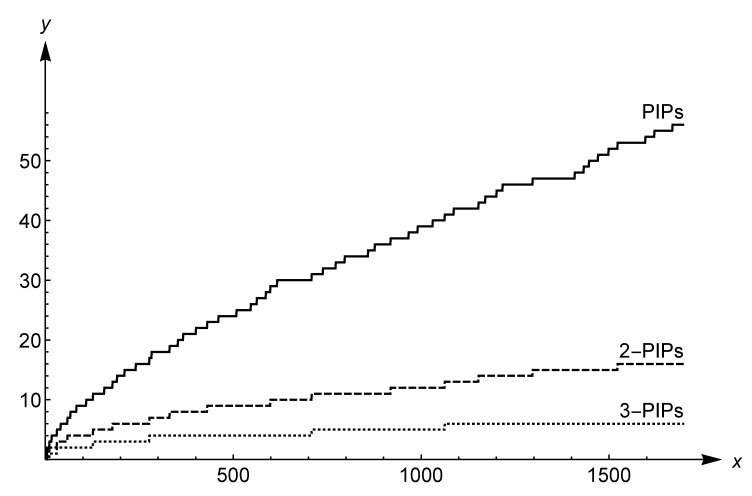
The behavior of *k*-order PIPs by the counting function $\pi^{k}$ for k=1,2,3.

The detailed proof can be found in [20]. Note that these bounds are certainly not the best possible. Nevertheless, the finding of better bounds seems difficult from any perspective and related studies are still lacking. Figure 3 shows the behavior of the *k*-order PIP counting function for k=1,2,3. According to the asymptotic equivalence (8), the family of distributions is strictly decreasing with the order *k*. Thus the natural question arises regarding the asymptotic comparison between *k*-order PIPs and Ramanujan primes. Let us denote the counting function of the Ramanujan primes with $\pi_{R}$. Note that the main difficulty in carrying out this issue is given by the asymptotic behavior of $\pi_{R}$. Sondow [17] and Shevelev [18] showed that

(9) $$\pi_{R}\left(x\right)∼\frac{x}{2\log x}∼\frac{\pi (x)}{2}\;\;\;\mathrm{as}\;\;x⟶+\infty \;.$$

**Figure 4:**
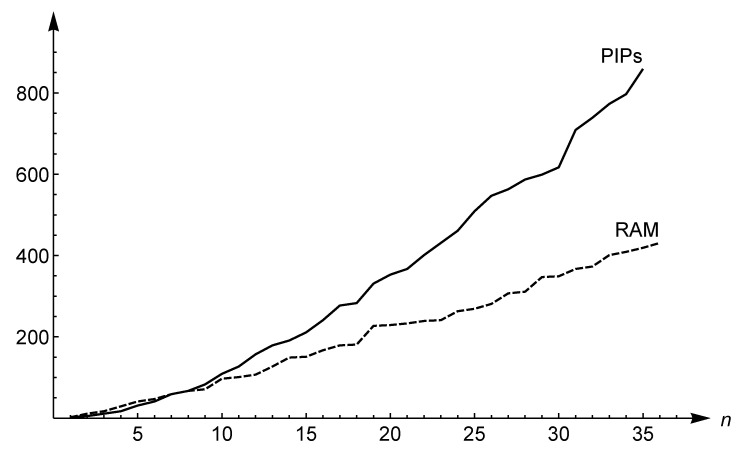
Comparison between PIPs and Ramanujan primes (RAM) in terms of cardinality. The first n=35 elements are depicted for both distributions.

Thus the comparison of asymptotic equivalences (8) and (9) assures us that the distribution of Ramanujan primes goes to infinity faster than the *k*-order PIP distribution. In addition to illustrating the asymptotic behavior, the comparison between *k*-order PIPs and Ramanujan primes for finite values of *x* is of interest. Fix a PIP *h* and consider the subset of $P_{1}$ given by H={3,5,11,…,h−1,h} of cardinality *n*. Figure 4 suggests that the PIP distribution increases more slowly than that of Ramanujan primes, except the values n≤8. All simulations have confirmed this assertion up to n=1500. Clearly, this is only an empirical clue and far from being a conclusive proof. Summarizing, we may formulate the following

**Conjecture** **1.** *The distribution of Ramanujan primes increases faster than the k-order PIP distribution except for the first eight elements.*

The main difficulty with any eventual proof of Conjecture 1 arises from the lack of results on both PIPs and Ramanujan primes. Note that it has to be based on analytic techniques and will probably appear in a forthcoming publication.

## 4. An Application in Dynamical Systems: The Hénon Map

**Figure 5:**
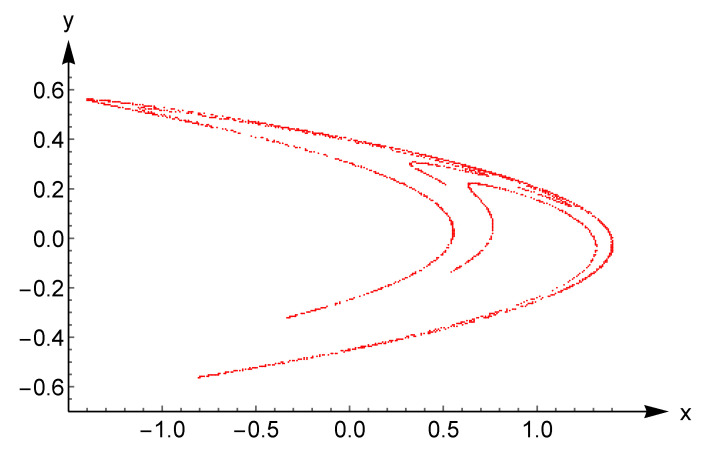
Iterative plot for the Hénon map with a=1.2, b=0.4, and 3000 iterations.

The results given in Section 3 provide an application in dynamical systems. In the literature, the link between dynamical systems and fractal geometry is widely investigated and discussed (see for instance [27] (Ch. 13)). According to Section 3, several studies show the central role of the Cantor set not only in fractal geometry but also in pure and applied mathematics. Likewise, the Hénon map *H* plays an important role in the theory of dynamical systems. In particular, it is defined as the family of maps $H\;:\;R^{2}\to R^{2}$ of the form

$$H\left(x,y\right)=\left(1+y-ax^{2},bx\right)\;,$$

where *a* and *b* are two real parameters such that a>0 and |b|<1 [28]. Each detailed analysis of *H* involves several issues and exceeds the scope of this paper. The major drawback occurs in the variation of *a* and *b*, which may entail sudden changes in behavior (bifurcations). The Hénon map shows periodicity, mixing, and sensitivity to initial conditions. Hénon dealt with the chaotic behavior of *H* for a=1.4 and b=0.3, called canonical parameter values for the Hénon map. This is illustrated in Figure 5. A multifractal study of *H* based on the Rényi dimension and the Lyapunov exponent was recently presented in [29]. For b=0.3 the simulation results indicate the convergence speed of orbits to the attractor for a∈[1,1.4]. Messano et al. [30] showed that all the aperiodic orbits can be removed from the dynamics of *H*, thus making the new dynamical systems globally convergent.

Among all the dynamical systems, the Hénon map has a close link with the Cantor set. The map *H* can of course be decomposed into an area-preserving bend, a contraction, and a reflection. The contraction suggests the presence of a fractal-like set. In fact, several simulations indicate that the Hénon map is locally the product of a line segment and a Cantor-like set *F* [27] (p. 196). Numerical results have clearly shown that the box-counting dimension of the attractor is about 1.26 for the canonical parameter values [29]. Summarizing, the simulation results mean simply that there are smooth bijections ϕ such that

(10) ϕ:[0,1]×F→B,

where *B* are small neighbourhoods of the attractor. The results given in Section 3 suggest that *F*, being a Cantor-like set, may be characterized by a suitable subset of prime numbers. The proof of this conjecture exceeds the scope of this paper. Nevertheless, let us now mention two important consequences. First, note that the role of prime numbers in the theory of chaotic dynamical systems might not be limited to only the description of periodic orbits, becoming a noteworthy parameter of their chaoticity. Second, and perhaps more importantly, the Cantor set and its generalizations are likely to play an increasingly relevant role in number theory, according to recent results [13,15,16].

## 5. Conclusions

This paper has investigated the link between fractal geometry and prime numbers. In particular, the study set out to find further confirmation of the fractal nature of prime numbers, as suggested in [10,13,15,16]. The proposed method is based on image analysis. The paper deals with the application of the binary image to three different topics (coprimality, PIPs, and Ramanujan primes), which play a central role in the theory of prime numbers. In all these cases the results, showing a fractal-like behavior, bear out the fractality of prime numbers. In fact, the coprimality condition depicts a binary image based on the Minkowski island. Likewise, the distribution of Ramanujan primes resembles the Cantor dust. In both the above cases, the lack of some symmetries might indicate the presence of randomness. Thus both the binary images may be seen as random fractals. Recently, Cattani and Ciancio [16] have shown that the PIP distribution looks like the Cantor dust. Accordingly, the Cantor set seems to play a relevant role in the hidden structure of prime numbers. In addition, the comparison between *k*-order PIPs and Ramanujan primes is discussed and the main conclusions are drawn.

The fractal-like behavior of prime numbers enables us to provide an application in chaotic dynamical systems. Being locally given by the smooth bijections (10), that is, by a Cantor-like set, the Hénon map may be described in terms of prime numbers. Therefore the chaoticity of several dynamical systems could be characterized by a suitable subset of P. Further research should be done to build the binary image of other prime subsets (Mersenne primes, twin primes, etc.). Clearly, several other questions remain to be addressed. In particular, the major drawback of this approach is that all these numerical results will require a theoretical proof. 

## Figures and Tables

**Table 1:**
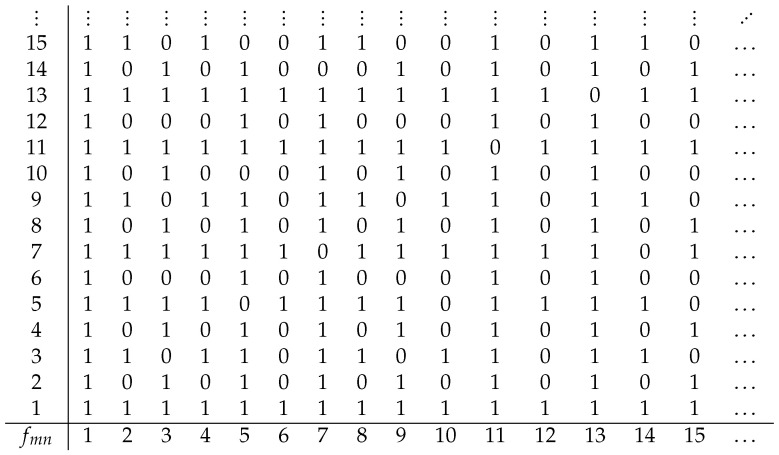
Correlation matrix for coprimality U(k×k) with 1≤k≤15.
